# A multimodal comparison of latent denoising diffusion probabilistic models and generative adversarial networks for medical image synthesis

**DOI:** 10.1038/s41598-023-39278-0

**Published:** 2023-07-26

**Authors:** Gustav Müller-Franzes, Jan Moritz Niehues, Firas Khader, Soroosh Tayebi Arasteh, Christoph Haarburger, Christiane Kuhl, Tianci Wang, Tianyu Han, Teresa Nolte, Sven Nebelung, Jakob Nikolas Kather, Daniel Truhn

**Affiliations:** 1 Department of Diagnostic and Interventional Radiology, University Hospital Aachen, Aachen, Germany; 2 Department of Medicine III, University Hospital Aachen, Aachen, Germany; 3 Ocumeda GmbH, Munich, Germany; 4 Else Kroener Fresenius Center for Digital Health, Technical University Dresden, Dresden, Germany

**Keywords:** Medical research, Translational research

## Abstract

Although generative adversarial networks (GANs) can produce large datasets, their limited diversity and fidelity have been recently addressed by denoising diffusion probabilistic models, which have demonstrated superiority in natural image synthesis. In this study, we introduce Medfusion, a conditional latent DDPM designed for medical image generation, and evaluate its performance against GANs, which currently represent the state-of-the-art. Medfusion was trained and compared with StyleGAN-3 using fundoscopy images from the AIROGS dataset, radiographs from the CheXpert dataset, and histopathology images from the CRCDX dataset. Based on previous studies, Progressively Growing GAN (ProGAN) and Conditional GAN (cGAN) were used as additional baselines on the CheXpert and CRCDX datasets, respectively. Medfusion exceeded GANs in terms of diversity (recall), achieving better scores of 0.40 compared to 0.19 in the AIROGS dataset, 0.41 compared to 0.02 (cGAN) and 0.24 (StyleGAN-3) in the CRMDX dataset, and 0.32 compared to 0.17 (ProGAN) and 0.08 (StyleGAN-3) in the CheXpert dataset. Furthermore, Medfusion exhibited equal or higher fidelity (precision) across all three datasets. Our study shows that Medfusion constitutes a promising alternative to GAN-based models for generating high-quality medical images, leading to improved diversity and less artifacts in the generated images.

## Introduction

The performance of deep learning crucially depends on the size of the available training set^1,2^. However, accessing large and diverse medical datasets can be challenging due to privacy concerns and limited data availability. To overcome these problems, generative adversarial models (GANs) have been utilized in the medical domain^3^. The applications of GANs are numerous, ranging from addressing legal or ethical challenges in data sharing^4^ to reducing data requirements through modality translation^5^ and improving deep learning performance^4,6^. However, generating meaningful medical data is hard, since medical diagnosis often depends on subtle changes in the appearance of complex organs and it is often more challenging than image classification on natural images. In addition, GANs suffer from inherent architectural problems such as the failure to capture true diversity, mode collapse, or unstable training behavior^7^. Thus, particular emphasis needs to be put on the generation of high-quality synthetic medical data.

Recently, denoising diffusion probabilistic models (DDPMs)^8^ and latent DDPMs^9^ have shown state-of-the-art results and were able to outperform GANs on natural images^10^. Yet, a wide-scale direct comparison of latent DDPMs to GANs on medical images covering multiple domains has so far not been performed.

However, some first studies compared DDPMs and GANs for medical image synthesis in specific use cases. Pinaya et al.^11^ used a latent DDPM to generate 3D brain MRI images. Their latent DDPM outperformed Least Squares GAN and Variational Autoencoder GAN. In a similar study by Dorjsembe et al.^12^, a DDPM was used to generate 3D brain MR images. In a quantitative comparison, the DDPM outperformed a 3D-α-Wasserstein GAN but not a Cycle Consistent Embedding GAN. Akbar et al.^13^, found that a DDPM tended to memorize the training images more than a StyleGAN when generating 2D brain MR images. When training a classification network with real or synthetic chest radiographs, Packhäuser et al.^14^ achieved a competitive area under the receiver operating curve (AUROC) with images generated by a latent DDPM, but not with those generated by a Progressively Growing GAN (ProGAN). Moghadam et al.^15^ demonstrated that a DDPM had superior performance to ProGAN for histopathological images. Kim et al.^16^ trained and compared ProGAN with a DDPM to generate synthetic fundus photographs with a resolution of 128 × 128 pixels. However, limited computational resources prevented them from training and evaluating their DDPM on images with 256 × 256 pixels or higher resolution. These studies established DDPMs as a promising alternative to GANs in the medical domain. However, only Pinaya et al. and Packhäuser et al. used latent DDPMs. Unlike conventional DDPMs, latent DDPMs allow generating larger images or 3D volumetric data^17^ due to sampling in the compressed latent space, which is particularly interesting for medical data.

In this study, we propose Medfusion, a conditional latent DDPM for medical image generation. We compare our DDPM-based model against GAN-based models by using images sourced from ophthalmology, radiology and histopathology and demonstrate that DDPMs beat GANs in terms of precision and diversity. To foster future research, we make our model publicly available as open-source to the scientific community.

## Results

### High reconstruction capacity of Medfusion’s autoencoder

Since the quality of latent diffusion-generated images is limited by the reconstruction quality of the autoencoder, we first investigated the reduction in the image quality caused by Medfusion’s autoencoder. To evaluate the maximum possible reconstruction quality, samples in the reference batches were encoded and decoded by the autoencoder. Subsequently, the Multiscale Structural Similarity Index Measure (MS-SSIM), mean squared error (MSE), and Peak Signal-to-Noise Ratio (PSNR) between the input images and reconstructed images were calculated and averaged over all samples (Table 1). All three metrics indicated a nearly perfect (MS-SSIM = 1, MSE = 0, PSNR ~ 40) reconstruction of the images in the AIROGS and CheXpert dataset. Reconstruction quality in the CRCDX dataset was good but lower, most likely due to the four times higher resolution of the input images. Since these metrics were measured on the reference set which is part of the training set, these values can be considered as an upper bound (for MS-SSIM and PSNR) and lower bound (for MSE), respectively. The results on the publicly available test set of the CheXpert and CRCDX dataset were, however, nearly identical to the results from the reference set and are available in the Supplemental Materials.

**Table 1:** Numerical evaluation of Medfusion’s autoencoder reconstruction quality.

	AIROGS	CRCDX	CheXpert
MS-SSIM	0.981 ± 0.007	0.901 ± 0.040	0.994 ± 0.001
MSE (x $1{0}^{-5}$)	11 ± 7	541 ± 305	25 ± 9
PSNR	40 ± 2	24 ± 3	36 ± 2

Values represent mean ± standard deviation.

*MSE* mean squared error, *MS-SSIM* multiscale structural similarity index measure, *PSNR* peak signal-to-noise ratio.

This experiment demonstrated that the autoencoder architecture of Medfusion did not restrict the image quality of synthesized images in terms of numeric metrics.

### Dataset-specific reconstruction challenges

**Figure 1:**
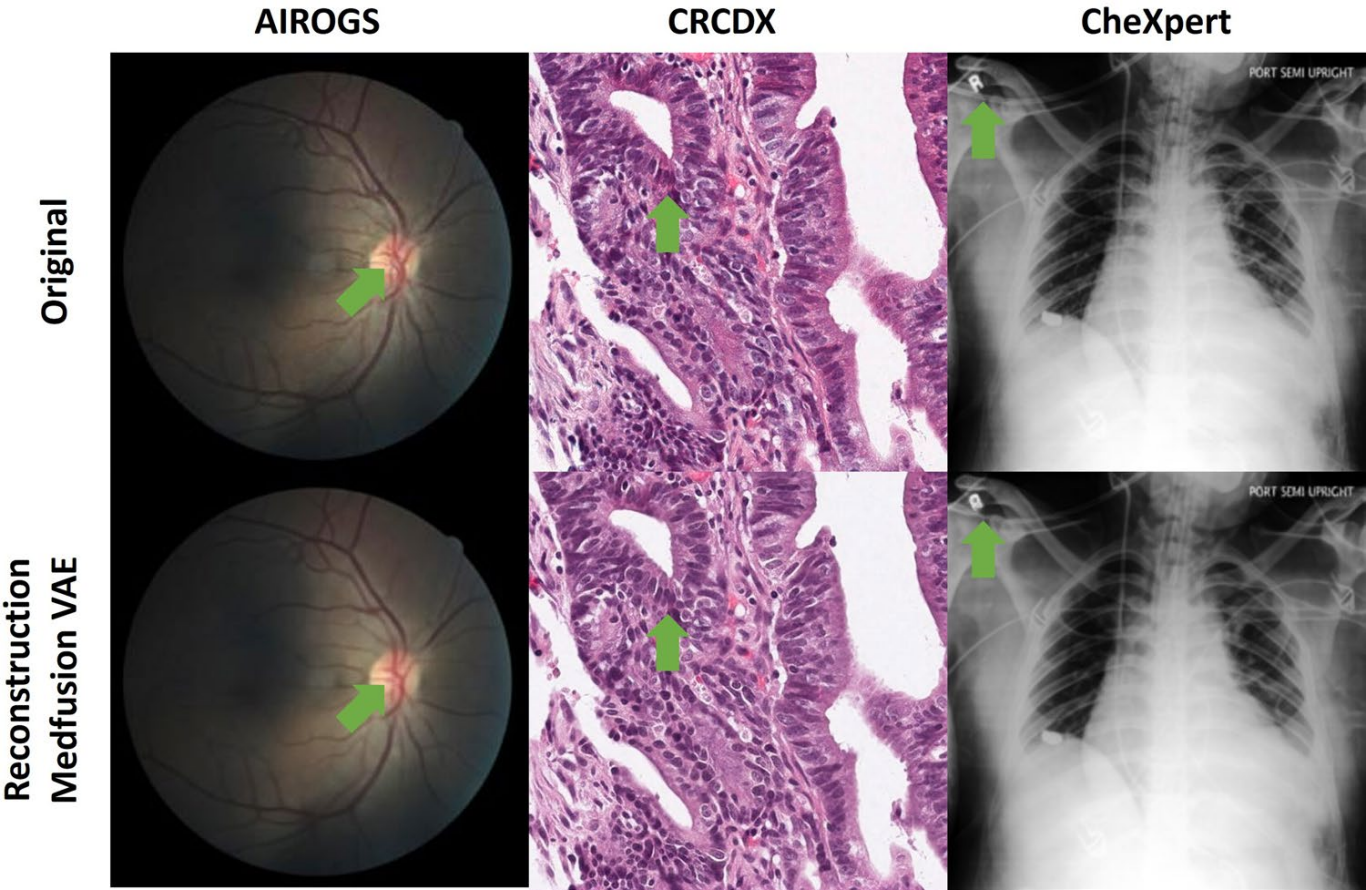
Reconstruction quality of Medfusion Variational Autoencoder (VAE). Original images (first row) and reconstructed images (second row) by the VAE in the AIRGOS, CRCDX, and CheXpert dataset. In the eye fundus images, fine deviations from the original images were apparent in the veins of the optical disc (green arrow). Slight deviations in the color tone (green arrow) could be observed in the CRCDX dataset. In the CheXpert dataset, letters (green arrow) became blurry after reconstruction.

To qualitatively investigate if autoencoding process led to reconstruction errors, we visually compared 50 original and reconstructed images side-by-side. This overall confirmed the numerically measured high reconstruction quality but revealed some dataset-specific reconstruction errors (Fig. 1). The compression in the autoencoding stage resulted in subtle structural changes in the fundus images, color changes in the histology images, and a loss of sharpness in the thorax images. This demonstrated that Medfusion’s image quality could be further enhanced by making use of a better autoencoding architecture. Therefore, we performed an additional experiment:

### Comparison with stable diffusion’s autoencoder

**Figure 2:**
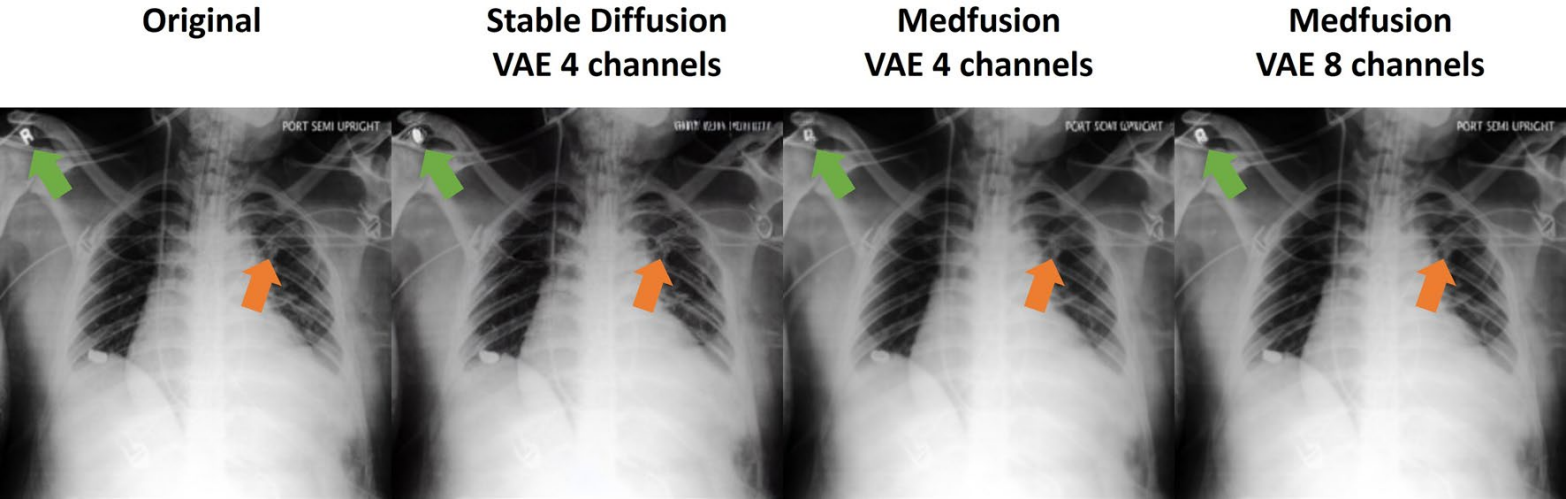
Reconstruction quality comparison. Both the “out-of-the-box” VAE of the Stable Diffusion Model (pre-trained on natural images, using four channels) and Medfusion’s VAE (trained on medical images, using 4 channels) produce artifacts that may affect diagnostic accuracy. In these image examples, lead cables were not reconstructed properly. Increasing the number of channels to 8 led to a more accurate reconstruction of such small structures by Medfusion.

A comparison with the autoencoder taken out of the box from the Stable Diffusion Model demonstrated that the reconstruction of medical images works well with an autoencoder pre-trained on natural images (Table 2). However, when comparing images side-by-side, Stable Diffusion’s autoencoder showed characteristic reconstruction errors in the CheXpert dataset when Stable Diffusion’s default variational autoencoder (VAE) with 4 channels was used (Fig. 2). Although less severe, reconstruction errors were also evident in Medfusion's VAE reconstructions. A further increase in the number of trainable parameters did not seem reasonable because Stable Diffusion 4-channel VAE already had about three times as many parameters as Medfusion’s 4-channel VAE (24 million). Therefore, we used 4 instead of 8 channels for Medfusion’s VAE, which resulted in a notable quality gain at the cost of compression ratio in the CheXpert dataset (Fig. 2).

**Table 2:** Numerical evaluation of Stable Diffusion’s autoencoder reconstruction quality.

	ARIOGS	CRCDX	CheXpert
MS-SSIM	0.973 ± 0.010	0.870 ± 0.049	0.973 ± 0.006
MSE ($1{0}^{-5}$)	22 ± 10	640 ± 383	90 ± 30
PSNR	37 ± 2	23 ± 3	30 ± 2

Values represent mean ± standard deviation.

*MSE* mean squared error, *MS-SSIM* multiscale structural similarity index measure, *PSNR* peak signal-to-noise ratio.

### Medfusion demonstrates superiority over GANs

**Figure 3:**
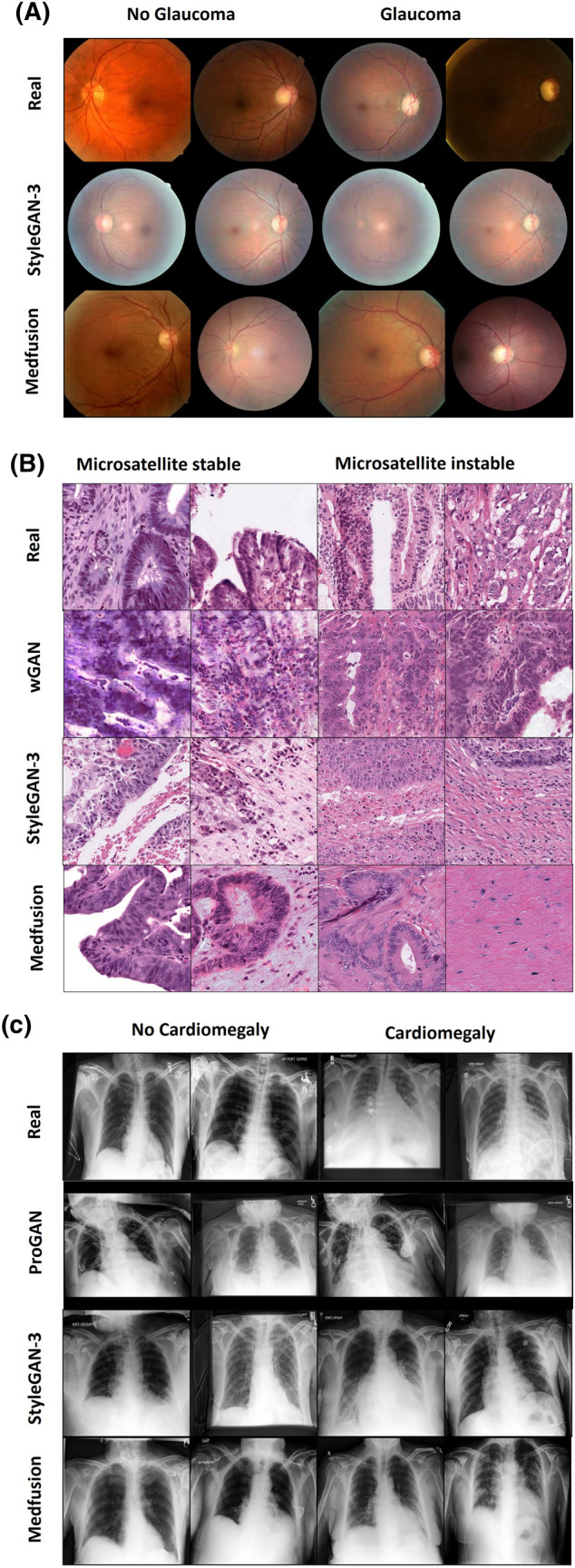
Qualitative image generation comparisons. Real images (first row), GAN-generated images (second row) and Medfusion-generated images (third row). Columns 1–2 and 3–4 show generated images for the labels no glaucoma and glaucoma (**A**), microsatellite stable and microsatellite instable (**B**) and no cardiomegaly and cardiomegaly (**C**), respectively.

When comparing real and synthetic images based on the Fréchet inception distance (FID) and Kernel inception distance (KID) metrics, we found that Medfusion generated more realistic-looking images (i.e., lower FID and KID; *P* < 0.001) in the AIROGS and CheXpert datasets compared to the GAN models (Table 3). Only in the CRCDX dataset, StyleGAN-3 exhibited a lower FID and KID, indicative of more realistic-looking images, compared to Medfusion (*P* < 0.001). Sample images for qualitative comparison are given in Fig. 3. Precision and recall values demonstrated that Medfusion achieved higher fidelity (*P* < 0.001, except for CRCDX *P* = 0.62) while preserving greater diversity (*P* < 0.001) in comparison to the GAN models. We visualize the overlap between the real and synthetic feature space using Principal Component Analysis (Supplementary Fig. S3).

**Table 3:** Quantitative image generation comparisons.

Dataset	Model	FID ↓	KID ↓	Precision ↑	Recall ↑
AIROGS	StyleGAN-3	20.43	0.019	0.43	0.19
	Medfusion	11.63	0.008	0.70	0.40
CRCDX	cGAN	49.26	0.036	0.64	0.02
	StyleGAN-3	18.83	0.014	0.57	0.24
	Medfusion	30.03	0.021	0.66	0.41
CheXpert	ProGAN	84.31	0.127	0.30	0.17
	StyleGAN-3	28.69	0.032	0.68	0.08
	Medfusion	17.28	0.020	0.68	0.32

Models include Generative Adversarial Networks (StyleGAN-3, cGAN, and ProGAN) and our proposed Medfusion model. Metrics for the best-performing model are indicated in bold.

*FID* fréchet inception distance, *KID* kernel inception distance.

Overall, the classification performance using the synthetic data was consistently lower than the real data, indicating that none of the generative models fully captured the relevant medical features (Table 4). However, Medfusion exhibited higher areas under the ROC curve (AUROC) values than the GAN models. Specifically, in the AIROGS dataset, Medfusion achieved a mean AUROC value of 0.88 compared to StyleGAN-3's AUROC value of 0.85 (*P* = 0.18). In the CRXDX dataset, Medfusion achieved a mean AUROC value of 0.57 compared to cGAN’s 0.50 (*P* = 0.01). In the CheXpert dataset, Medfusion achieved an AUROC value of 0.77 compared to ProGAN’s 0.74 (*P* = 0.36).

**Table 4:** AUROC of ResNet when trained on real data or on synthetic data generated by the GANs or Mefusion.

	Real data	GAN (previously proposed)	GAN (StyleGAN-3)	Medfusion
AIROGS	0.89	N/A	0.85	0.88
CRCDX	0.63	0.50	0.45	0.57
CheXpert	0.85	0.74	0.73	0.77

Based on previous studies, the cGAN was used for the CRCDX and ProGAN for the CheXpert dataset while no previous GAN study was available for AIROGS (N/A).

Based on the quantitative and qualitative results, we concluded that DDPM generated more realistic and diverse synthetic images than GANs in all investigated medical domains.

We provide a website with sample images to the scientific community so that a straightforward and more comprehensive review of Medfusion’s image quality is possible. The website can be accessed at: https://huggingface.co/spaces/mueller-franzes/medfusion-app.

### GAN-Synthesized images exhibit characteristic artefacts

**Figure 4:**
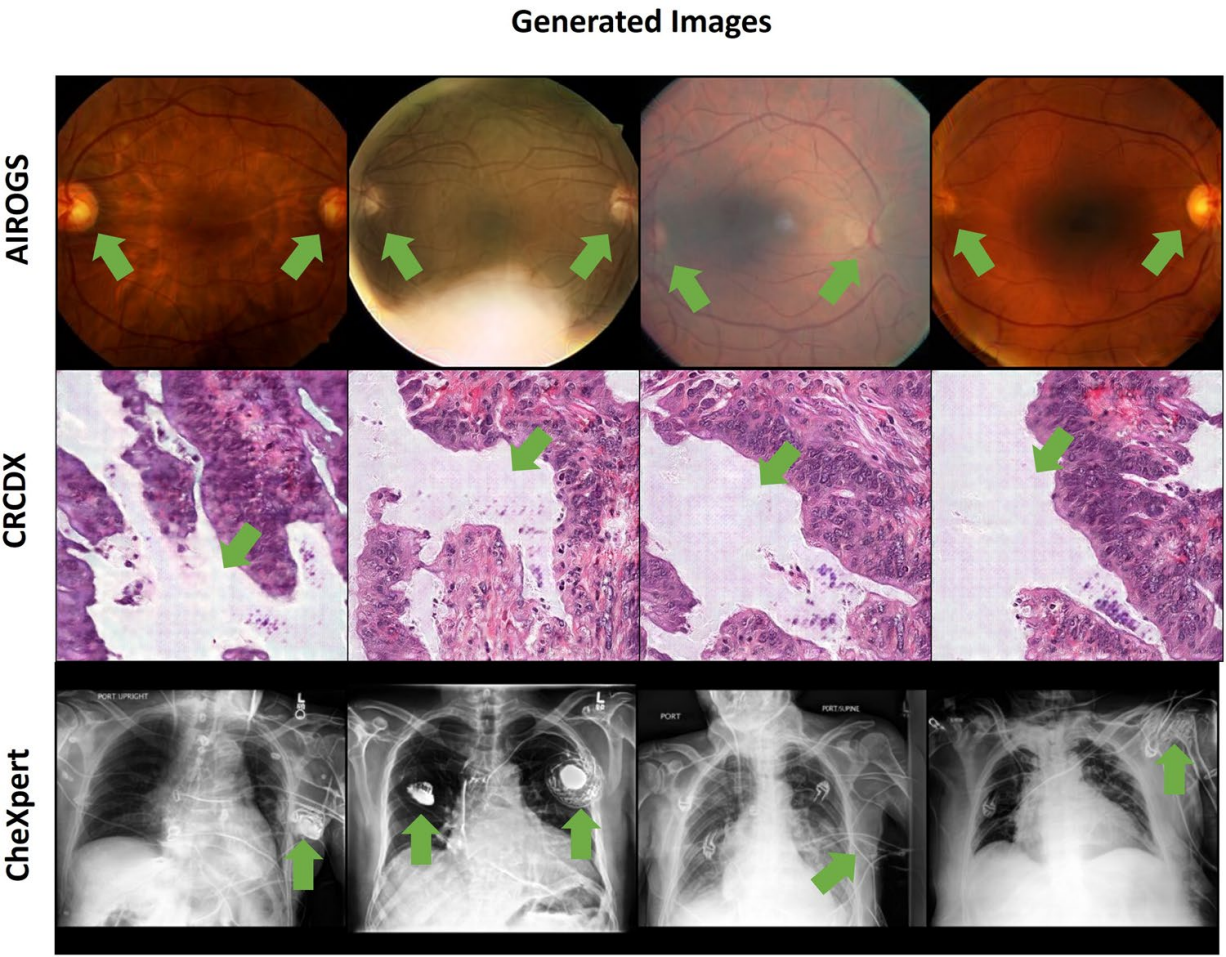
GAN-generated images that can be easily identified as synthetic. Synthetic images generated by StyleGAN-3 were easily identifiable because of two optical discs in eye fundus images (first row), artificial grid patterns in cGAN generated histology images (middle row), and fuzzy borders and irregular appearances of medical devices (model independent) in chest X-ray images (bottom row).

Characteristic visual artifacts were noted for the GAN-synthesized images (Fig. 4). For the eye fundus images, we found that the synthetic image sometimes exhibited two optical discs, while every real fundoscopy always only exhibits one optical disc. No such occurrences were noted for the Medfusion-generated images. The cGAN-generated images exhibited an artificial grid pattern for some generated histological images. We did not observe such artifacts for the Medfusion or StyleGAN-3 model. Chest radiographs were identifiable as synthetic by blurred letters or fuzzy borders and irregular appearances of medical devices (e.g., cardiac pacemakers). We found these artifacts to appear in both the GAN-generated and Medfusion-generated synthetic images. It should be noted that some of the real images showed strong visual artifacts due to image acquisition errors. However, the real artifacts differed from the artifacts in the synthetic data. Examples of such artifacts are provided in the Supplementary Material.

## Discussion

The success of Deep Learning depends largely on the size and quality of training data. Therefore, generative models have been proposed as a solution to extend the availability of training data^4^. DDPMs have been demonstrated to achieve superior image quality on natural images. In this study, we investigated if such models can also generate more diverse and realistic images as compared to GAN-based models in the medical domain.

We explored DDPM in three domains of medical data: ophthalmologic data (fundoscopic images), radiological data (chest X-rays) and histological data (whole slide images of stained tissue). We optimized our Medfusion architecture for medical image synthesis and found that image quality of DDPM-generated images was superior to that of the baseline GAN-generated images: Medfusion achieved an FID score of 11.63 in the eye, 30.03 in the histology, and 17.28 in the chest dataset which were all lower (better) than those of the GAN models (20.43, 49.26, 84.31; *P* < 0.001), indicating higher image quality. Also, the precision of the images generated by Medfusion was higher in the eye (0.70 vs. 0.43; *P* < 0.001), histology (0.66 vs. 0.64; *P* < 0.001), and chest (0.68 vs. 0.30; *P* < 0.001) dataset, indicating higher fidelity. A known problem with GANs is mode collapse, where the generator produces very realistic (high precision) but too similar images so that the true diversity between the real images is not represented. Recall, as a measure of diversity, was strikingly low for histological images generated by the cGAN compared to Medfusion (0.02 vs. 0.41; *P* < 0.001), which indicates a mode collapse. The low recall of the StyleGAN-3 in the CheXpert dataset also indicates a possible mode collapse.

In a study by Pinaya et al.^11^, a latent DDPM was trained to generate 3D MRI brain images and compared with two GANs. In agreement with our study, the latent DDPM model showed a lower (better) FID score of 0.008 compared to the two GANs (0.023 and 0.1576). Remarkably, FIDs were 3 to 4 orders of magnitude lower than in our study. We suspect that this is due to the 3D data used instead of 2D data because our measured FIDs are in the same order of magnitude as in previous studies on natural 2D images. Regardless of whether a GAN or our latent DDPM was used, we observed a maximum recall (diversity) of approximately 0.4 on the medical datasets. On natural images, recalls of 0.5 or better were observable^10^. One possible reason for this is that natural images can achieve an overall higher diversity by changing backgrounds and colors, medical images often have a constant (black or white) background, and colors are narrowly limited to e.g. grayscale. Therefore, diversity in medical images mainly manifests as changes in details (e.g., variations in heart size or in the opacity of lung tissue). Thus, it may be more difficult to achieve high diversity while maintaining high fidelity in medical image generation than in natural images. Future studies are needed to investigate this hypothesis.

Our study has limitations. Firstly, the training and generation of the CheXpert and AIROGS images were performed in a lower resolution than the original ones and the images were square (i.e. height equals width). This was done to stay consistent with the GAN results from previous studies, which were trained and evaluated for a specific lower resolution, and because StyleGAN-3 only allowed for a quadratic resolutions that are a power of 2. Future studies should investigate how the Medfusion model behaves for higher resolutions compared to GAN models.

Secondly, we would like to point out that the metrics used in this study to evaluate image quality were not developed for medical images in the first place, which means that they should in general be evaluated with care. The development of metrics that are proxies for human judgment is still an ongoing topic area of research^18^.

Thirdly, to keep our study focused, we have not performed an ablation study to investigate the specific contributions of different network components on the generated images. However, a more detailed analysis provides a deeper understanding and needs to be addressed in future research.

Our study demonstrated that latent DDPMs are promising alternatives for medical image generation besides GANs because they demonstrated higher diversity, fidelity, and classification performance of diseases in three different modalities. By incorporating diverse GAN architectures, including StyleGAN-3, our study provides a comprehensive analysis that goes beyond previous studies. Finally, by employing a latent DDPM instead of a conventional DDPM, our study showed that the generation of high-resolution medical images becomes feasible.

## Materials and methods

### Ethics statement

All experiments were conducted in accordance with the Declaration of Helsinki and the International Ethical Guidelines for Biomedical Research Involving Human Subjects by the Council for International Organizations of Medical Sciences (CIOMS). The study has additionally been approved by the local ethical committee (EK 22-319).

### Datasets

In this retrospective study, three publicly available datasets were used.

First, the AIROGS^19^ challenge train dataset, containing 101,442 RGB eye fundus images from about 60,357 subjects of which 98,172 had “no referable glaucoma” and 3270 with “referable glaucoma”. Sex and age of the subjects were unknown. All images were scaled to 256 × 256.

Second, the CRCDX^20^ dataset, containing 19,958 color-normalized 512 × 512 RGB histology colorectal cancer images at a resolution of 0.5 µm/px. Half of the images were microsatellite stable and microsatellite instable, respectively. Sex and age of the subjects were unknown.

Third, the CheXpert^21^ train dataset, containing 223,414 Gy-scaled chest radiographs of 64,540 patients. Images taken in lateral position were excluded, leaving 191,027 images from 64,534 patients. All images were scaled to 256 × 256 and normalized between -1 and 1, following the pre-processing routine in^4^. Of the remaining radiographs, 23,385 showed an enlarged heart (cardiomegaly), 7,869 showed no cardiomegaly, and 159,773 had an unknown status. Labels with unknown status were relabeled as in^4^ resulting in 160,935 images without cardiomegaly and 30,092 with cardiomegaly, respectively. Mean age of the 28,729 female and 35,811 male patients was 60 ± 18 years.

### Model architecture and training details

Two types of generative models were used in this study.

First, classical generative adversarial networks (GANs) as introduced by Goodfellow et al.^22^. We aimed to use GANs that previously exhibited state-of-the-art quality on the respective datasets to allow a fair comparison with the diffusion model.

For the CheXpert dataset, we used a pre-trained progressively growing GAN (proGAN) from^4^. In a previous study, chest X-rays generated by this GAN were barely differentiable by three inexperienced and three experienced radiologists^4^. Furthermore, proGAN has already been used for data augmentation and has led to higher downstream performance as compared to traditional augmentation^23^.

For the CRCDX dataset, we employed a pre-trained, conditional GAN (cGAN) as described in^6^. This GAN has been shown to produce realistic-looking histological cancer images in a blinded test with five readers and the authors were able to show that a classifier benefits from using generated images during training.

No pre-trained GAN was available for the AIROGS dataset at the time of writing. Therefore, StyleGAN-3^24^ was used as the current top-performing and state-of-the-art GAN architecture. We also trained StyleGAN-3 on the CheXpert and CRCDX datasets to demonstrate its performance against ProGan and cGAN models, as well as to provide a comparison between the latest developments in the field of generative models. We used the default settings proposed by the authors of the StyleGAN-3 publication for images of 256 × 256 and 512 × 512 pixels, i.e., a batch size of 32, snap equals 10 and gamma equals 2 or 8, respectively. The model was trained until the FID did not decrease for three consecutive epochs. This condition was met after 1000 and 3000 training steps, respectively.

**Figure 5:**
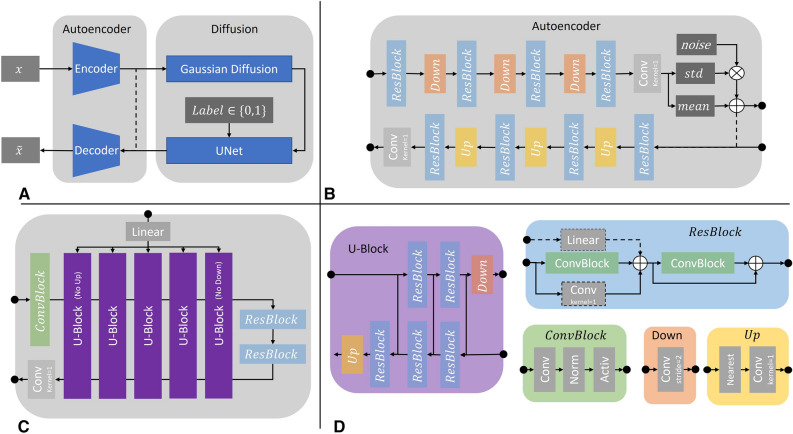
Illustration of the Medfusion model. (**A**) General overview of the architecture. $x$ and  $\widetilde{x}$ are the input and output images. (**B**) Details of the autoencoder with a sampling of the latent space via the reparameterization trick at the end of the encoder and a direct connection (dashed lines) into the decoder (only active for training the autoencoder). (**C**) Detailed view of the denoising UNet with a linear layer for time and label embedding. (**D**) Detailed view of the submodules inside the autoencoder and UNet. If not specified otherwise, a convolution kernel size of 3 × 3, GroupNorm with 8 groups, and Swish activation was used.

Second, our proposed Medfusion model (Fig. 5), that is based on the Stable Diffusion model^9^. It consists of two parts: an autoencoder that encodes the image space into a compressed latent space and a DDPM^8^. Both parts were trained subsequently. In the first training phase, the autoencoder was trained using the Adam optimizer^25^ with a learning rate of 0.001. It encoded the image space into an 8-times compressed latent space of size 32 × 32 and 64 × 64 for the 256 × 256 and 512 × 512 input space, respectively. During this training phase, the latent space was directly decoded back into image space and supervised by a multi-resolution loss function, which is further described in the Supplemental Material. In the second training phase, the pre-trained autoencoder encoded the image space into a latent space, which was then diffused into Gaussian noise using t = 1000 steps. A UNet^26^ model was trained using the AdamW optimizer^27^ with a learning rate of 0.001 to denoise the latent space. The L1 loss was used to supervise the difference between the real and estimated noise distribution. During this training phase, the weights of the autoencoder were frozen. Images were generated with a Denoising Diffusion Implicit Model (DDIM)^28^ and t = 150 steps. We motivate our choice of steps in the Supplemental Material.

## Experimental design

The study was divided into two sub-studies.

First, we investigated whether the capacity of the autoencoder in the Medfusion model was sufficient to encode the images into a latent, highly compressed space and decode the latent space back into the image space without losing relevant medical details. It was also investigated whether the autoencoder of the Stable Diffusion Model (pre-trained on natural images) could be used directly for medical images, i.e. without further training on medical images and loss of medically relevant image details.

Second, we compared the images generated by Medfusion and the GANs quantitatively and qualitatively. For the quantitative evaluation, we would like to refer to the statistics section in which we go into detail about the individual metrics. For the qualitative assessment, 50 real, GAN-generated, and Medfusion-generated images were compared side-by-side by G.M.-F. and D.T.

### Statistical analysis

All statistical analyses were performed using Python v3.8 and the TorchMetrics library^29^.

To compare sample quality between models, the following metrics were used. First, the Fréchet Inception Distance (FID)^30^ was implemented, which is now considered the standard metric for quality comparisons of generative models^18^ and measures the agreement of the real and synthetic images by comparing the features of the deepest layer of the Inception-v3^31^ model. Second, the Kernel Inception Distance (KID)^32^ was used, which aims to improve the FID by using a polynomial kernel function to compare the real and synthetic image features and eliminates the strict Gaussian assumption. Third, the Improved Precision and Recall^33^ metric was performed, which measures the fidelity of the generated samples as the overlap of synthetic and real features relative to the entire set of synthetic features (precision) and the diversity as the overlap relative to the entire set of real features (recall). Following a previous study^10^, Inception-v3 was used instead of the original proposed VGG-16^34^ to extract features. Of note, the used metrics depend on the reference subset and implementation^35^ and are not directly comparable with other studies. To ensure consistency between model comparisons, a reference batch was used for the AIROGS, CRCDX, and CheXpert dataset with 6540, 19,958, and 15,738 equally distributed images of both classes.

To assess how well the datasets’ classes were represented and important medical imaging features were preserved, we trained a ResNet classification model using real or synthetic data. Synthetic data were generated by the distinct GAN architectures or our latent DDPM model (Medfusion). Detailed information regarding the training process can be found at “Classification on Real or Synthetic Images” in the Supplementary Material.

To evaluate the image quality after compression by the autoencoder, we utilized the following metrics.

First, we calculated the Multiscale Structural Similarity Index Measure (MS-SSIM)^36^, which is a generalized version of the SSIM^37^ by applying SSIM at different resolutions. The SSIM measures image distortion by the structural information change expressed by comparing luminance, contrast, and structure. Second, we calculated the Mean Squared Error (MSE) to quantify the average squared difference between the original and reconstructed images. It provides a measure of overall pixel-level dissimilarity. Third, we calculated the Peak Signal-To-Noise Ratio (PSNR), which compares the maximum possible signal (power) to the power of the noise in the reconstructed image.

Permutation tests were used to calculate p-values. We intentionally chose not to define a specific significance level to prevent the dichotomization of our results as either significant or non-significant^38^.

### Implementation

All experiments were implemented in Python v3.8 and were executed on a computer with an Nvidia RTX 3090. The datasets can be downloaded directly from the websites of the referenced authors. Source code for the StyleGAN-3, ProGAN, and cGAN are available at https://github.com/NVlabs/stylegan3, https://github.com/peterhan91/Thorax_GAN and https://github.com/mjendrusch/pytorch-histogan. For reproducibility, we publish our code and model weights as parts of this paper at https://github.com/mueller-franzes/medfusion under an open-source license.

## Supplementary Information

Supplementary Information.

## Data Availability

The datasets generated during and/or analysed during the current study are available from the corresponding author on reasonable request. The AIROGS dataset is available at https://zenodo.org/record/5793241#.Y-zHbHbMKbg, the CRCDX at https://zenodo.org/record/3832231#.Y-zHuXbMKbg and the CheXpert at https://stanfordaimi.azurewebsites.net/datasets/8cbd9ed4-2eb9-4565-affc-111cf4f7ebe2.
