# Supplementary material for: A multimodal comparison of latent denoising diffusion probabilistic models and generative adversarial networks for medical image synthesis
Source: Sci Rep. 2023 Jul 26;13:12098. doi: 10.1038/s41598-023-39278-0 (PMC10372018; doi:10.1038/s41598-023-39278-0)
Supplement: Supplementary file 1 — Supplementary Information. [file 41598_2023_39278_MOESM1_ESM.docx]

# Supplemental Material

### Artefacts in Real Data

Sifting through about 300 images of the AIROGS, CheXpert, and CRCDX data set, several images could be identified that showed strong visual artifacts (**Figure 1**). Types of artifacts were limited to those that are severe and make the images appear "unnatural" because they are rare in the clinical routine or are even specific to the three data sets.


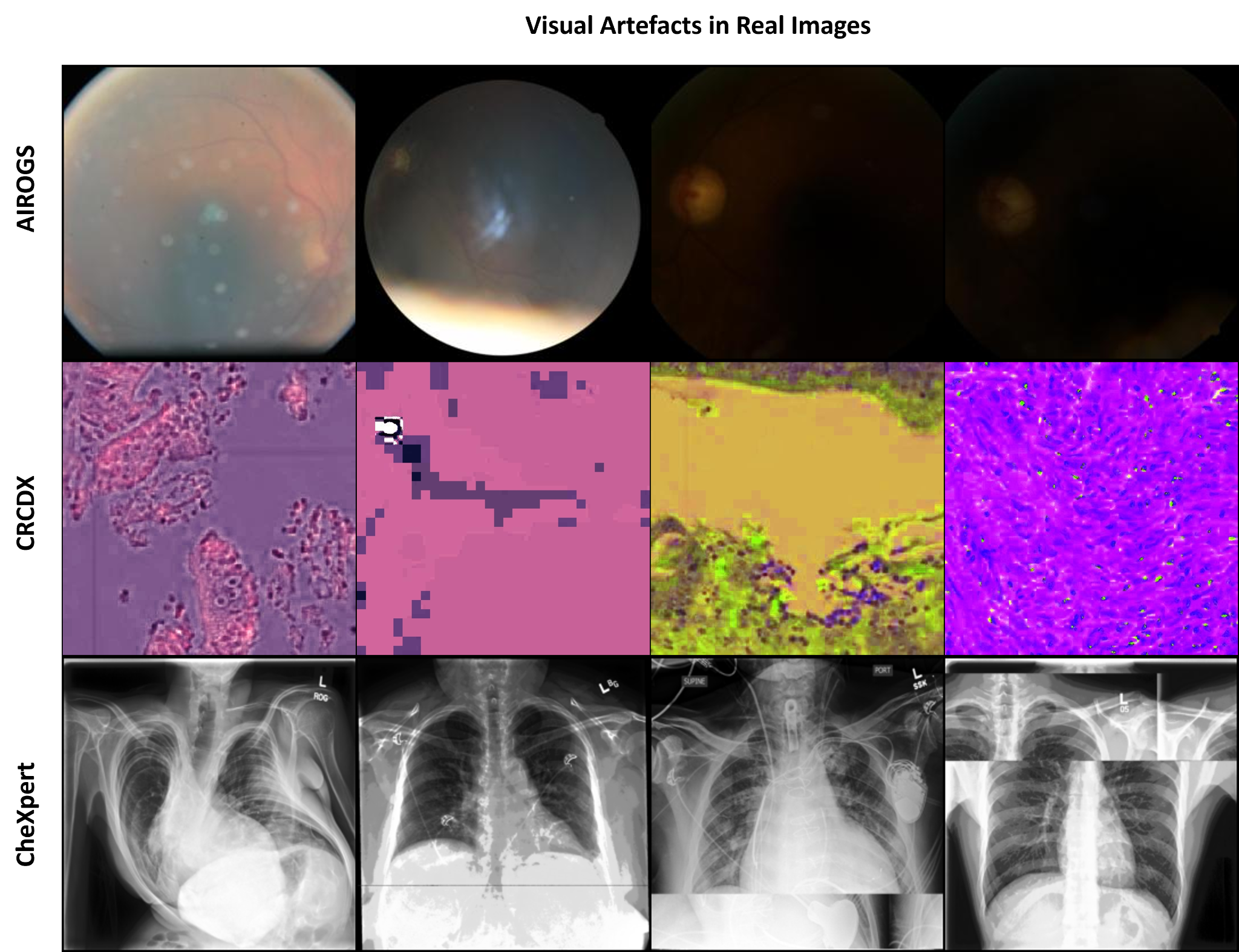


**Figure 1: Examples of visual artifacts in the ARIOGS, CRCDX, and CheXpert datasets.**

### Autoencoder Loss

The autoencoder loss consisted of an embedding loss and a reconstruction loss.

We employed the Kullback–Leibler divergence between the embedding space and white Gaussian noise as embedding loss. The reconstruction loss was calculated between the input image and reconstruction output image as the summation of absolute distance (L1), Learned Perceptual Image Patch Similarity (LPIPS) (30), structural similarity index measure (SSIM) (28), and PatchGAN discriminator (31).

Besides the final, full-resolution prediction, the first-lower resolution of the decoder was also supervised by the reconstruction loss. Note that by applying SSIM to multiple resolutions, it becomes MS-SSIM (27) and the multi-resolution PatchGAN resembles the multi-resolution loss of Pix2PixHD (32).

### Autoencoder Testset

The variational autoencoders of Stable Diffusion and Medfusion were evaluated on a test set independent of the training set (**Table 1**). The CheXpert test set included 200 frontal chest X-rays (134 without, 66 with cardiomegaly) and the CRCDX test set included 32,361 histology images (5,223 microsatellite unstable, 27,138 stable). A public test set for the AIROGS dataset did not exist.

**Table 1**: **Autoencoder reconstruction quality.**

|  | CRCDX (Testset) | | CheXpert (Testset) | |
| --- | --- | --- | --- | --- |
|  | Stable Diffusion | Medfusion | Stable Diffusion | Medfusion |
| MS-SSIM ↑ | 0.876±0.045 | 0.903±0.037 | 0.974±0.006 | 0.995±0.001 |
| MSE ($10^{-5}$) ↓ | 617±404 | 528±316 | 89±28 | 24±8 |

Values represent mean±standard deviation, MSE = Mean Squared Error, MS-SSIM = Multiscale Structural Similarity Index Measure

### DDIM Sampling Steps

We increased the number of sampling steps of Medfusion's DDIM from t=50 to 250 in the inference mode and measured FID, precision, and recall on the reference data set (**Figure 2**). In terms of the three metrics, there was an increase in image quality with increasing number of steps. In general, quality increased notably in the first 150 steps and then reached a plateau. Therefore, 150 steps appeared to be an appropriate tradeoff between globally increasing quality and increasing inference time.

###


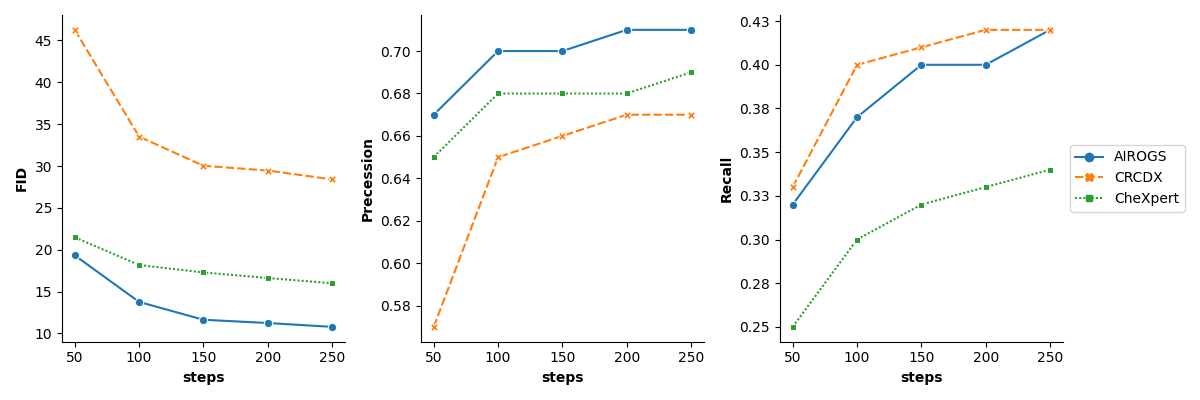


**Figure 2: Fréchet Inception Distance (FID), Precision, and Recall as a function of the sampling steps.**

### Classification on Real or Synthetic Images

We utilized a ResNet-101 (40) architecture as our classifier. The input data comprised either real or synthetic images, partitioned into training and validation sets with an 80/20 split ratio. The binary cross entropy loss function was employed during training. We terminated the training process if the loss function did not exhibit a further decrease for a continuous span of 30 epochs. The model yielding the best performance during training was evaluated on a distinct test subset comprising real data exclusively. For optimization, we employed the AdamW optimizer with a learning rate set to 0.0001.


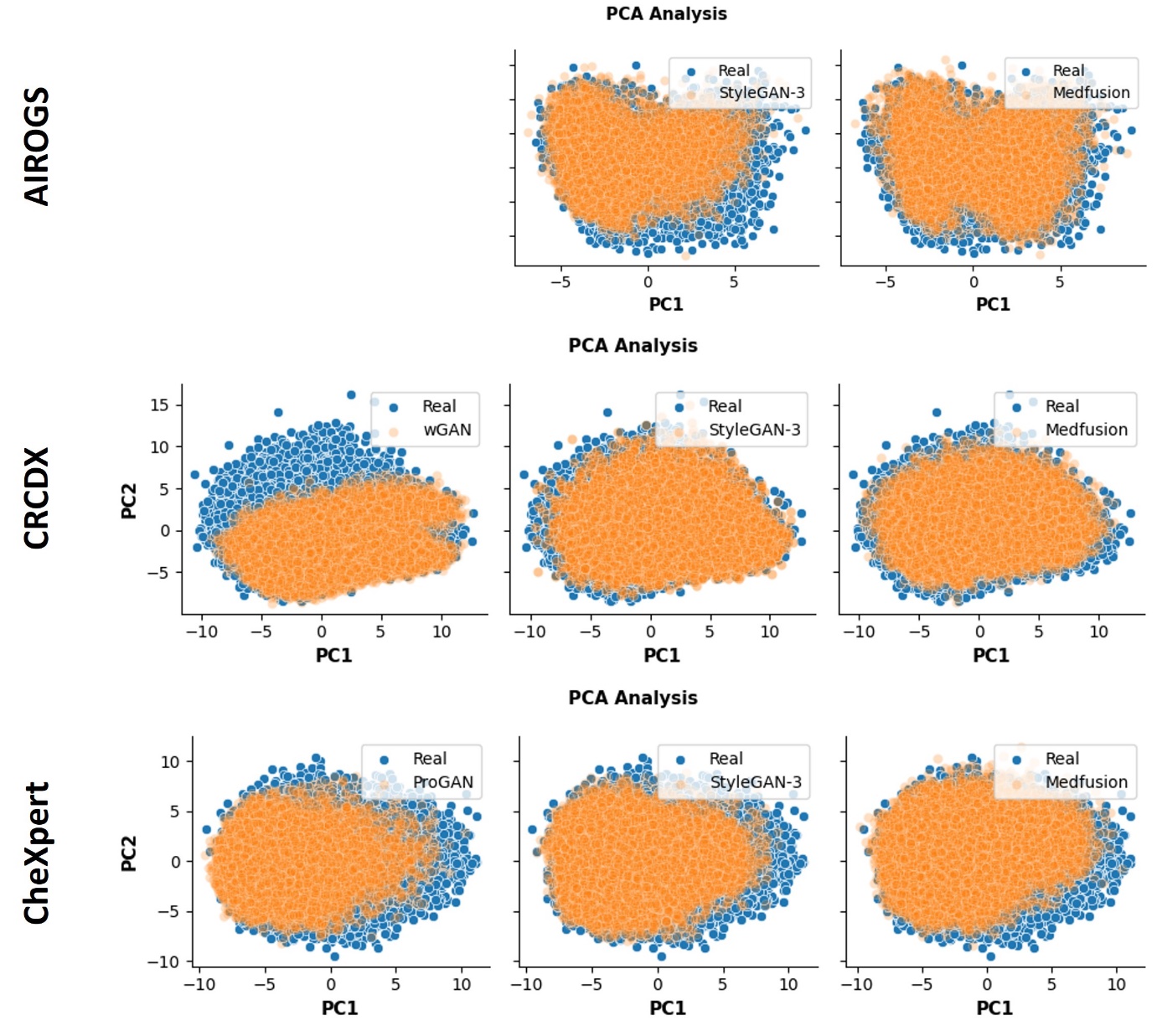


**Figure 3: Visualization of the real and synthetic data distribution using a PCA analysis.**
